# Establishment and Characteristic Analysis of a Dog Model for Autologous Homologous Cranioplasty

**DOI:** 10.1155/2020/5324719

**Published:** 2020-05-22

**Authors:** Wenyu Zhu, Jie Wu, Haifeng Zhao, Weihua Wang, Lichun Lu, Ke Yan, Yin Yin, Qiang Huang

**Affiliations:** ^1^Department of Neurosurgery, The Affiliated Suzhou Science & Technology Town Hospital of Nanjing Medical University, Suzhou, Jiangsu, China; ^2^Institute of Clinical Medicine Research, The Affiliated Suzhou Science & Technology Town Hospital of Nanjing Medical University, Suzhou, Jiangsu, China; ^3^Department of Pathology, The Affiliated Suzhou Science & Technology Town Hospital of Nanjing Medical University, Suzhou, Jiangsu, China; ^4^Laboratory Animal Center, Soochow University, Suzhou, Jiangsu, China; ^5^Department of Neurosurgery, The Second Affiliated Hospital of Soochow University, Suzhou, Jiangsu, China

## Abstract

**Objective:**

The aim of this study is to establish a large animal (dog) model that can be referred clinically for autologous homologous cranioplasty.

**Methods:**

Our large skull defect dog model was established by emulating the decompressive craniectomy with 22 adult beagle dogs. The autologous bones were taken out from the dogs and divided into two groups, the freeze-drying (FD) group and the single freezing (SF) group. They were then stored in the bone bank at -20°C after being irradiated with 25 KGy. Three months later, the bones were reimplanted. After operation, we closely watch the experimental objects for four more months examining the infection and survival of the bone graft.

**Results:**

Through macroscopic observation, it was found that, among 44 cranial flaps (bilateral) from the rest of the 22 dogs, grade A cranial flaps accounted for 86.4% (19/22) in the SF group and only 31.8% (7/22) in the FD group. Although osteogenic osteoclast, Harvard tube, neovascularization, and angiogenic factors were found through the pathological results, including an electron microscope and calmodulin tracer, it could be verified by using X-CT and micro-CT that early bone resorption could be still found even in grade A bone flap.

**Conclusion:**

By using the common clinical method to preserve the cranial flaps, we established an experimental dog model of autologous cranioplasty for a large area of cranial defect. It was proved that this model could reproduce the infections and bone resorption which typically happened in clinical autologous homologous cranioplasty. As a conclusion, the established model can be used as an effective experimental tool for further research to improve the success rate of autologous homologous cranioplasty.

## 1. Introduction

Decompressive craniectomy is often performed to alleviate intracranial pressure caused by craniocerebral injury, cerebral hemorrhage, etc. However, the resulted cranial defects need a secondary operation, i.e., cranioplasty [1]. Initially, silicone rubber was used as the repairing material. Later on, three-dimensional titanium mesh, which is constructed through CT graphic analysis, has become more and more popular. However, due to subcutaneous fluid infection, scar contraction, and slow healing, titanium mesh may get exposed. As a result, the patient may face the risk of removing it. More importantly, titanium's attributes of heat conduction, hardness, and anti-impact are obviously worse than those of autologous cranial flaps [2, 3]. Autogenous cranioplasty was performed very early in clinical practice. Because of its excellent biocompatibility, relatively strong anti-infective ability, no risk of disease transmission, and low cost, autogenous cranioplasty should have been the first choice [4–6]. Unfortunately, due to severe bone resorption and unpredictable infection occurring from time to time, it has never been widely used in China, with only a few sporadic reports in recent years [7, 8]. To solve these problems and promote autogenous cranioplasty, we believe that it is necessary to establish an effective big animal model. As we know, in order to increase the survival rate for patient with cerebral hernia, the large bone flap decompression, i.e., extreme craniotomy, has become a clinical standard. Therefore, the animal model used for emulating large skull defects should also be based on the extreme craniotomy of large animals. There are quite a few reports about extreme craniotomy based on rabbits, while those on dog models are very limited [9], even though the latter ones are obviously closer to the clinical practices [10]. In this paper, a large animal model is established based on beagle dogs. Bilateral extreme craniotomy was carried out for each dog, and autologous cranioplasty was performed after 3 months. Both of the preservation of bone flaps and the reimplantation followed the clinical process. It was shown that the infection and bone absorption happening in clinical cranioplasty can be easily reproduced. The detailed model establishment and analysis of infection and bone resorption are as follows.

## 2. Materials and Methods

### 2.1. Extreme Craniotomy to Remove the Bone Flap

**Figure 1:**
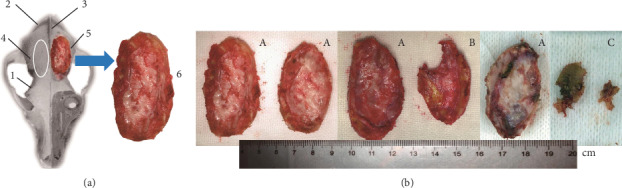
Schematic diagram and evaluation quality for the harvesting of dog cranial flap. (a) Schematic diagram for the removal of the dog cranial flap. 1: temporal line; 2: occipitoparietal suture; 3: external sagittal crest; 4: the model of the removed cranial flap; 5: cranial flap to be harvested; 6: enlarged picture of harvested cranial flap. (b) Evaluation of bone graft appearance quality: grade A, relatively complete with even thickness; grade B, incomplete with defect area ≤ 30%; grade C, defect area > 30%.

Beagle dogs aged approximately 2 years and weighing approximately 10 kg of both genders were purchased from Shanghai Jiagan Biotechnology Co., Ltd. (license No.: SYXK (Shanghai) 2013-0087). They were raised at the Center for Experimental Animals of Suzhou University (license No.: SYXK (Jiangsu) 2012-0062). The operation permit was issued by the Animal Ethics Committee of Suzhou University. The craniotomies were carried out following the clinical requirements of the large cranial flap removal. After intravenous injection of 2.5% pentobarbital sodium at 1 ml/kg, an oropharynx tube was placed to keep the respiratory tract unobstructed. Then, one piece of cranial flap with the size of 2.5 cm × 4.5 cm was extracted from each side of the front temporal parietal region of the dog with the cranial drill and scalpel designed for neurosurgery (Aesculap AG, Germany) (Figure 1(a)). With the dura mater unopened, the muscles and scalp were sutured layer by layer after full hemostasis. The animals were taken care of until awake. To prevent infection, 1.6 million units of penicillin was intramuscularly injected before the surgery. The same intramuscular injection of penicillin was provided after the surgery twice a day for continuously three days.

### 2.2. Storage of Cranial Flaps

The removed cranial flaps were cleaned in sterile saline and stored in sterile containers, which were sent to the biological tissue aseptic system (model ATC610, developed by Suzhou Aoteran Medical Technology Inc.) for packaging. The processed cranial flaps were then divided into two groups, the FD group and the SF group, respectively. The FD groups were frozen and dried according to the freeze-drying curve, where the water content was kept within the range of 2~6% and stored in a -20°C refrigerator for 48~72 h after sealing. The SF group went through the same process only without being frozen and dried. Both groups were irradiated with 25 KGy under full frozen chain and then stored in a bone bank at -20°C.

### 2.3. Reimplantation of Cranial Flaps

After 3 months, the containers with dog cranial flaps were taken out from the bone bank and thawed in sterile saline at room temperature for 12 hours. The rest of the processes followed the neurosurgical requirements of autologous homologous cranioplasty. After general anesthesia, the periosteum and the dura mater were separated along the original incision to the margin of the bone window. The cranial flaps were then reimplanted to the original position, followed by stratified suturing of the muscle layer, fascia, and scalp. Animals were nursed until recovery and then kept in cages. Same as previously mentioned, antibiotic injections were provided to prevent infections.

### 2.4. Examination of Reimplanted Cranial Flaps

By scanning the living dogs with medical CT (Siemens, Germany), the CT bone window images of the dog model reimplanted bone were obtained, and the corresponding bone mineral density was measured. In addition, the osteogenesis of the reimplanted bone was examined by using calcein (Sigma, USA) fluorescence tracer. Starting from the day of reimplantation, calcein with the dose of 5 mg/kg was injected subcutaneously every two weeks for 6 times. After 4 months, the experimental dogs were killed and the implant bones together with the original skull were taken out by cutting through 1 cm beyond the edge of the reimplanted bone graft. Without decalcification, the specimens were made according to the requirements of hard tissue slices. The combination of calcein and active calcium was examined through a fluorescence microscope (Zeiss, Germany). Other tests were also performed as follows. (1) Pathological examination: samples were collected at the center and edge of the cranial flaps and also at the junction of the new and old bones. After routine decalcification, paraffin embedding, slicing, H&E staining (Baso, China), as well as immunohistochemical staining with CD31 (Biorbyt, China, 1 : 300) and CD34 (Abcam, USA, 1 : 250), they were further examined with an optical microscope. (2) Bone mineral density measurement: micro-CT (SkyScan1176, Belgium) was used to scan the fresh cranial flaps, and the data were analyzed by CTAN software. (3) Ultrastructure analysis of the transmission electron microscope (Hitachi7700, Japan): the new bone and the old bone were cut into small bone slices with the size of about 1 square mm; from which, the specimens were made according to the requirements of the transmission electron microscope for the ultrastructure examination.

### 2.5. Statistical Analysis

The data were represented as mean ± SD and compared using one-way ANOVA analysis. Chi-squared tests were used to evaluate the cranial flap quality and incision healing. The statistical significance was set at *P* < 0.05 (^∗^*P* < 0.05; ^∗∗^*P* < 0.01; ^∗∗∗^*P* < 0.001).

## 3. Results

### 3.1. General Conditions of the Experimental Dogs and Aesthetic Evaluation of Bone Grafts

The experimental dog underwent three operations during 7 months. All surgical incisions were classified as a type I sterile wound. From the point of view of healing, the good healing incisions, the poor healing incisions, and the ones with infection or pus were classified as A, B, and C, respectively. The aesthetic quality of the reimplanted bones obtained from the third operation was also classified into three grades, i.e., A, B, and C, through macroscopic observation in natural light. In detail, the grade A implied that the bones were relatively uniform, complete, and close to those implanted through the second craniotomy. Grade B implied that the incomplete and defect area was ≤30%, or the thickness of the bone flap could be felt a little uneven by palpation. Grade C meant that the defect area was >30%, the thickness of the bone flap could be felt significantly uneven by palpation, or bones were replaced by soft connective tissues instead (Figure 1(b)) and could not even be felt. By using the *X*^2^ test, it was showed that the aesthetic quality had no significant difference for different grades of incision healing. However, significant difference could be found for different preservation methods (FD and SF) where *P* < 0.01 (Table 1). Among the 22 cranial flaps in the FD group, only 7 were grade A, while 15 were grade B or C. On the other hand, among the 22 cranial flaps in the SF group, 19 were Grade A, and only 3 were B or C. The results showed that the quality of cranial flaps in the SF group was better than that in the FD group.

### 3.2. Conventional and Molecular Histopathology of Reimplanted Bone

**Figure 2:**
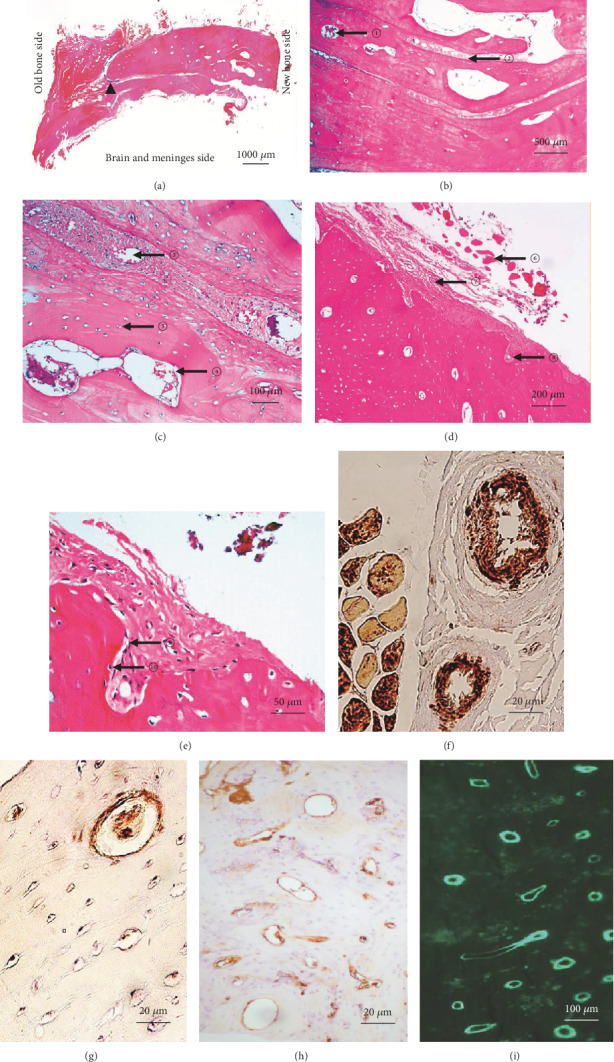
H&E staining and immunostaing of the reimplanted bone slices. (a) The overview of the sample, ▲ denotes the gap between old and new bones. (b-e) The bone marrow cavity with different sizes of blood cells ① and ④, diploe ②, and nourishing blood vessels within the diploe ③. The outermost surface of the skull was the muscle ⑥, followed by the fibrous tissue and periosteum ⑦. Some of the periosteum, together with the nourishing blood vessels, seem to have been embedded in the outer plate of the skull ⑧ and gradually transformed into osteoblasts ⑨ and osteocytes ⑩. A large amount of matured bone cells that are densely distributed can be seen as small dots in the red bone matrix ⑤. (f-g) The CD34-stained deep brown immune complexes deposited in the microbeam of muscle fiber ((f) left), the periosteal blood vessels ((f) right), the mesenchymal vascular in the bone tissue, the bone lacuna, and the trabecular vessels. (h) The CD31-stained brown immune complexes deposited in the mesenchymal vascular in the bone tissue, Haversian system, and trabecular vessels. (i) The bone trabecula composed of bone active calcium labeled by calcein, with morphology similar to that of some positive structures of CD34 and CD31.

Observed under optical microscopy, at the junction of the replanted bone (new) and the original cranial bone (old) at the edge of the defect window, there is a gap filled with periosteum and meninges, similar to the cranial suture (Figure 2(a)). Indeed, cranial sutures represent a functionally active unit that promotes osteogenesis, whereas suture-like junctions caused by surgery, especially those that are relatively loose, can be considered early bone resorption. (Figure 2 In Figure 2(a), the red stained part on the left side is obviously the old bone, while the light red part of the right side represents the new bone with incomplete osteogenesis. Furthermore, nourishing vessels, diploe bone marrow cavity, osteocytes, and other structures required for new bone formation can also been observed in the new bones (Figures 2(b)–2(e)). In particular, temporal muscle fiber bundles, the periosteum, bone matrix, and bone trabecula can be found in the SP immunohistochemical stained slices, which is also consistent with what was observed in H&E staining. In addition, the CD34+ or CD31+ immune complexes including the Haversian canal (Figures 2(f)–2(h)) further confirmed that these tissues did have regenerated blood vessels with different maturity.

### 3.3. Ultrastructure of Reimplanted Bone

**Figure 3:**
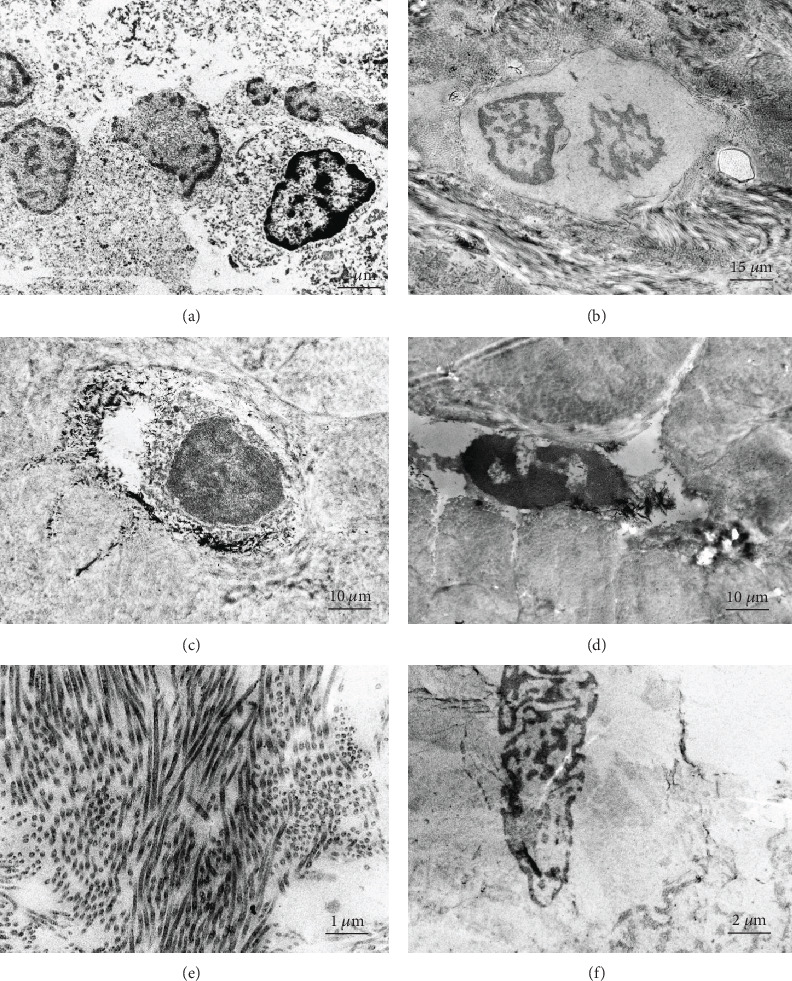
Transmission electron microscopy of reimplanted bone: (a) osteoblasts, (b) osteoclasts, (c) bone lacuna, (d) bone canalicules, (e) collagen fiber, and (f) fibroblasts.

In the transmission electron microscope analysis, multipoints were sampled for the new bone graft. Most osteocytes were typical osteoblasts, which had large nuclei, rich chromatin, and a relatively small nucleus-cytoplasmic ratio. Osteoclasts with two nuclei were also found occasionally (Figures 3(a) and 3(b)). Meanwhile, there were also bone lacunae containing osteocytes and bone canaliculus connecting the Haversian canal (Figures 3(c) and 3(d)). In addition, collagen fibers and fibroblasts distributed in dots or plexuses (Figures 3(e) and 3(f)) were quite common.

### 3.4. Fluorescent Imaging of the Reimplanted Bone

**Figure 4:**
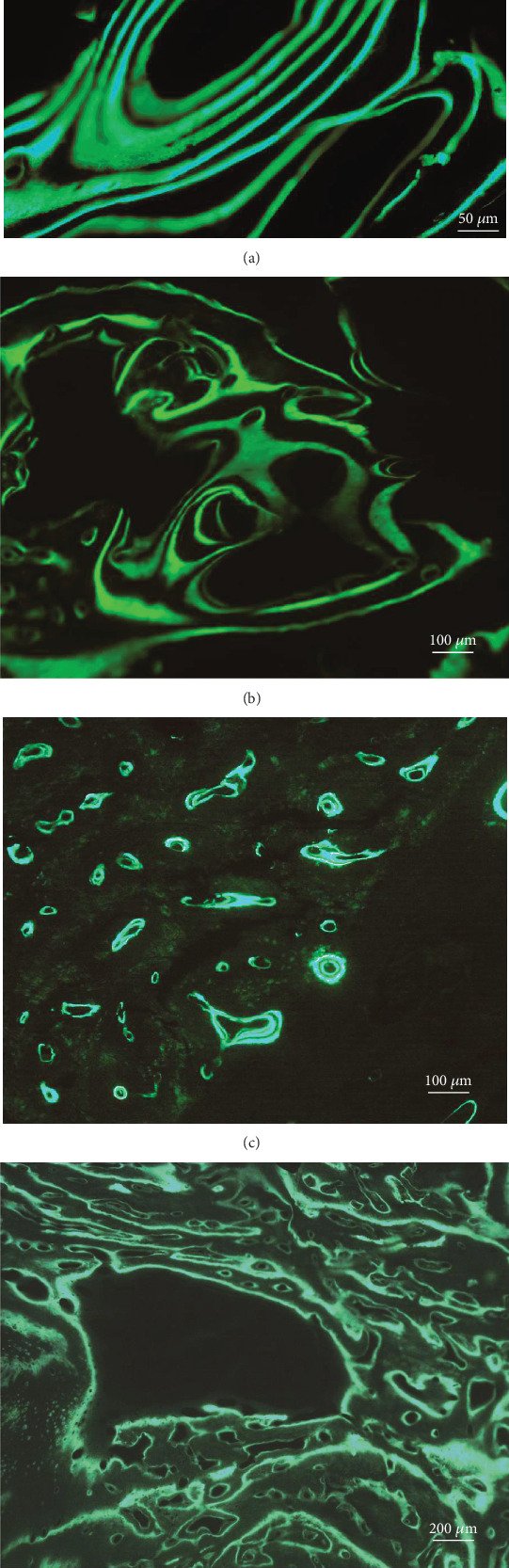
Calcein tracing in bone graft: under a fluorescence microscope, a new bone graft is combined with calcein and shows as bright green. It is deposited in the Haversian system of the bone tissue and the osteocytes. (a) Mature Harvard system. (b) Bone matrix. (c) Primary Harvard system. (d) Deposited active calcium.

Calcein glows in green with 470 nm excitation and 535 nm emission. It can be used to identify the deposit structure of the bone tissues. Those in the multiple concentric circles were typical Haversian systems, where the number of concentric circles was determined by the maturity of the corresponding Haversian canal. Those with only two circles were the early-stage Haversian canal (Figures 4(a) and 4(c)). Additionally, amorphous active calcium deposits were also found, which might be associated with the various new bones distributed in the bone matrix (Figures 4(b) and 4(d)).

### 3.5. X-CT and Micro-CT Imaging of the Reimplanted Bone

**Figure 5:**
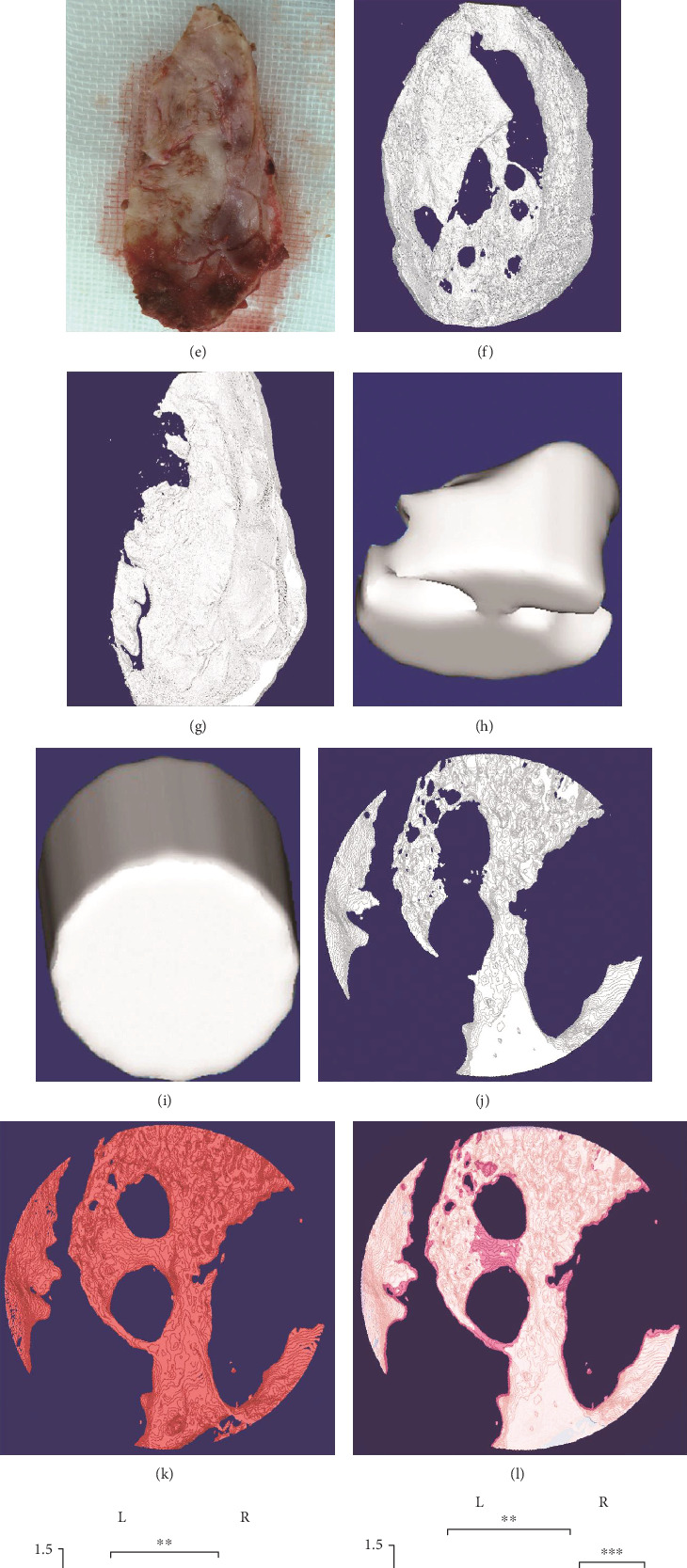
X-CT, micro-CT imaging, and statistical analysis. (a) Sagittal CT image. (b) Coronal CT image. The arrow in (a) is the bone defect at the reimplantation site. The arro in (b) is the reimplanted cranial flaps. (c-e) Detected samples. (f-g) 3D reconstruction of the whole bone flap. (h, i) 3D reconstruction of different points of a bone graft. (j–l) Pseudocolor map in the D region of interest ((j) reimplanted bone, (k) new bone, and (l) fusion of both). (m) One-way ANOVA analysis of the micro-CT data with the addition of the ratio of the left and right bone flaps. Here, L denotes the left, R denotes the right, OB denotes old bone, and NB denotes new bone.

Dogs were scanned with X-CT after anesthesia. The maturity of the new bone could be measured by CT density. Compared with the old bone, the CT density of the new bone was generally low, and some areas had only soft tissue shadow (Figures 5(a) and 5(b)). To select the cranial flaps removed during the third operation, Micro-CT was carried out on the sagittal and coronal planes in an area of 3 × 2 cm at a precision of 18 *μ*m, followed by 2D and 3D reconstruction of the obtained data. Furthermore, the CTAN software and the method proposed by Peyrin et al. were used to mark the red pseudocolor of the new bone to identify the positional relationship between the new bone and the reimplanted bone [11]. The results showed that the grades of reimplanted bones, which were assessed by macroscopic observation and palpation, were significantly different from those observed with micro-CT. Specifically, some cranial flaps, which were identified as grade A through macroscopic observation and palpation, were found uneven under the light. Moreover, micro-CT could identify the area that was lacking of bone composition (Figures 5(c)–5(g)), which was especially obvious in the 3D image of the new bone (Figures 5(h) and 5(i)). In the constructed pseudocolor image, the unabsorbed old bones were marked as gray color, the new bones were marked as red, and the junctional areas, where new bones were completely merged with the old bones, were marked as pink (Figures 5(j)–5(l)). Six points of the medial new bone and six points of the lateral old bone were selected from around the junction for comparison. The detailed ultrastructure analysis could be found in Table 2, where bone mineral density (BMD), BMD of tissue volume (BMD of TV), bone volume (BV), tissue volume (TV), and bone volume/tissue volume (BV/TV) were listed for comparison between new and old bones. Except for TV, the other 4 indicators showed significant difference between new and old bones under SPSS19 system (*P* < 0.001), which implies that the new bone was imperfect osteogenesis (Figure 5(m)).

## 4. Discussion

### 4.1. Situations and Challenges of Autologous Cranioplasty

According to the database of CNKI (China National Knowledge Infrastructure), 16,077 cases of autologous cranioplasty had been reported in 342 papers from 1975 to October 2017 in China. Other than China, according to the meta-analysis of Corliss et al. [12], there were 5,346 cases of autologous cranioplasty reported during the period of 1975~2015. In these studies, cryopreservation (CP) and subcutaneous abdominal implantation (AP) were the two major methods to preserve the cranial. The infection rate was 7.3% for CP and 7.1% for AP, respectively. The proportion of surgical correction due to unsatisfying cranioplasty was 15.9% for CP and 7.6% for AP, respectively. The proportion of reabsorption detected by physical examination was 9.7% for CP and 7.7% for AP, respectively. There seems to be no significant difference between the two preservation methods. After autologous cranial flap replantation, the adult bone resorption ratio was 3-22% [13–18]. Recent literature data suggest that this figure is more complex in children than the 50% complication rate initially described by De Bonis et al. and Frassanito et al. [18, 19]. Indeed, the risk of reabsorption appears to decrease linearly with age in the pediatric population [19]. Moreover, Herteleer et al., Piitulainen et al., and Lee et al. reported that severe complications, which required removal of reimplanted bone through a secondary operation after autologous cranioplasty, happened in 29.7% of 74 cases, 40% of 20 cases, and 44.4% of 18 cases, separately [20–22]. Therefore, although autogenous cranioplasty is promising in theory, it has not been widely used in clinic in China, as few hospitals are willing to take commercial risks arising from the storage of autogenous cranial flaps, including nonprofit and higher risk of bone resorption and infection [8].

### 4.2. Causes of Infection and Reabsorption in Cranial Flaps

The cause of cranial flap infection is complicated. Although all operating procedures at each step followed the requirement of sterility, the preservation of extracted cranial flaps and the following cranioplasty might still introduce contamination. Because the osteocytes and bone tissues of the autologous skull had to be kept unaffected [23], the cranial flaps could not be treated with extreme high-pressure sterilization as metal surgical instruments, which was noted in the Guidelines for Autologous Tissues Management. Presently, cryopreservation is the most widely used. According to the guidelines, samples should be preserved at -20°C for half a year or at -80°C for 5 years. However, Chan et al. reported that among the 38 cases of cranial flaps preserved at -80°C, 18 were cultured with osteocytes and bacteria, and all osteocytes did not survive. In addition, five cases (27.8%) were cultured with bacteria [24]. It was concluded that osteocytes could not survive more than 4 months of cryopreservation. On the other hand, Tahir et al. stated that there were only three cases (3.4%) of infection among 88 cases preserved at -20°C, and he believed that it was not necessary to preserve the samples with expensive cryogenic equipment [25]. As a conclusion, it cannot be confirmed that a lower preservation temperature of cranial flaps will help to lower the infection rate. In summary, although there are guidelines for reference, further explorations are still needed for the preservation of cranial flaps to prevent infections. For experimental convenience, the preservation temperature of -20°C in the special bone bank equipment was chosen in this paper.

Besides infection, reabsorption of the autologous skull after reimplantation was another important reason of failure and its happening rate varied in different studies. Schuss et al. considered cranial flap reabsorption (CFR) as a long-term complication after autologous cranioplasty [26]. He found that there was only 4% BFR in 254 cases within a year. However, in a retrospective analysis for 10 years, the incidence of severe complications was as high as 29.7% in 74 cases of autologous cranioplasty [20]. In detail, a test for bacteriology was first ordered before cranial flap preservation. Samples which tested positive were irradiated at 25~40 KGy and stored in the refrigerator at -80°C. Among the 38 cases preserved by this approach, 14 cases had bacteria detected again before reimplantation. After cranioplasty, no complete reimplanted bone could be found in any of them. Therefore, high-dose irradiation can neither sterilize completely nor maintain the activity of osteoblasts. In another word, it is hard to find effective preventive measures before the reabsorption mechanism of the reimplanted bone is identified. Our experiments also showed similar results. 15 of the 22 cranial flaps did not reach grade A in the FD group after being irradiated by 25 KGy. In addition, although the proportion of grade A reached 19/22 (86.4%, Figure 1) for the SF group, these cranial flaps were still found to be incompletely developed under X-CT and micro-CT (Figure 5). As a conclusion, the cranial flaps irradiated by 25 KGy could not have the activity of the original osteoblasts fully preserved for either group. Although irradiation can effectively reduce the immunogenicity, it is not necessary for autologous skull transplantation, where there is no immunological rejection. Therefore, it may not be appropriate to take high-dose irradiation for the sterilization and preservation of the bone flap.

### 4.3. Prospects

Currently, there is no universally acknowledged solution to the infection and bone absorption for autologous homologous cranioplasty, despite the guidelines from the United States [23]. The traditional irradiation and low temperature preservation may physically damage the bone cells and the molecular activity of protein. There is no consensus yet on the optimal temperature, which is used to prevent infection and maintain the activity of cranial flaps. So far as we know, a lower temperature does not imply a better result. Herteleer et al. suggested that bone resorption was a normal physiological phenomenon in autologous bone healing, and it happened in up to 90% of cases [20]. However, this conclusion came from the retrospective analysis using bioinformatics R language, which had not been confirmed by clinical prospective studies. Andy mentioned that macrophages played an important role in fracture healing. The interaction between mesenchymal stem cells and macrophages is closely related to bone healing [27]. Since osteoclasts are indispensable in the process of bone resorption, some studies suggest that inhibiting the formation of osteoclasts may significantly reduce bone loss [28, 29]. So, we believe that the control of local environmental, including macrophages, may play an important role after reimplantation. If the imbalance of mutual restriction between osteoclasts and osteoblasts is the major cause of bone resorption, inhibiting macrophage-induced hyperfunction of osteoclasts will be a good strategy to solve the problem. Due to the clinical limitation, further research can be carried out on our established model to verify this hypothesis.

Considering the establishment of a more simulated animal model, the disadvantage of this model is that the dural is intact when the cranial flap is removed, which is inconsistent with the actual clinical situation. In fact, the dura is still very helpful to repair the damaged skull [30]. Therefore, in the future, it is necessary to establish an animal model of skull flap decompression in accordance with basic diseases (such as brain trauma) and on this basis to carry out autogenous skull transplantation research.

## Figures and Tables

**Table 1:** Grafted cranial flap grouping and rating statistics (*n* = 22).

No.	1	2	3	4	5	6	7	8	9	10	11	12	13	14	15	16	17	18	19	20	21	22
GP	FD	FD	FD	FD	SF	SF	SF	FD	FD	SF	SF	SF	SF	FD	FD	FD	FD	SF	SF	SF	SF	FD
BFPT	12	12	12	12	12	12	12	6	6	6	6	6	6	3	3	3	3	3	3	3	3	12
OP1	a	b	a	a	b	b	a	Cc	a	a	a	b	a	b	a	a	a	a	a	a	a	a
OP2	a	b	a	a	c	a	a	a	a	a	C	a	b	a	a	b	a	a	a	a	a	a
NBFR-L	B	C	B	A	A	A	A	C	B	A	C	A	B	B	C	C	A	A	A	A	A	A
NBFR-R	B	C	B	A	A	A	A	C	B	A	C	A	A	A	C	C	A	A	A	A	A	A

Note: No. denotes the number of dogs; GP denotes the experimental grouping; FD denotes the freeze-drying group; SF denotes the single freezing group; BFPT denotes the time of preservation in vitro (month); OP1 denotes the rating of the first surgical incision; OP2 denotes the rating of the second surgical incision; NBFR-L and NBFR-R denote the ratings of the left and right reimplanted cranial flaps, respectively; the dogs who died incidentally during the experiment were excluded from the rating.

**Table 2:** Grafted cranial flap micro-CT analysis of BMD, BMD of TV, BV, and BV/TV (*X* ± *s*, *n* = 6).

	Left			Right		
	Old bone	New bone	*P*	Old bone	New bone	*P*
TV	0.95435 ± 0	0.95435 ± 0	*P* < 0.001	0.95435 ± 0	0.95435 ± 0	*P* < 0.001
BMD	0.9303 ± 0.3926	0.3508 ± 0.1553	*P* < 0.001	1.0649 ± 0.0460	0.3260 ± 0.1480	*P* < 0.001
BMD of TV	0.9390 ± 0.0393	0.3508 ± 0.1553	*P* < 0.001	1.0645 ± 0.0461	0.3261 ± 0.1480	*P* < 0.001
BV	0.9362 ± 0.0147	0.1691 ± 0.1250	*P* < 0.001	0.9478 ± 0.0118	0.2148 ± 0.1248	*P* < 0.001
BV/TV	98.1031 ± 1.5436	16.9604 ± 13.7491	*P* < 0.001	99.3154 ± 1.2376	22.5090 ± 13.0812	*P* < 0.001

Note: BMD: bone mineral density (cm^2^/g); BMD of TV: bone mineral density of tissue volume (g/cm^3^); BV: bone volume (mm^3^); BV/TV: bone volume/tissue volume (%).

## Data Availability

The data used to support the findings of this study are included within the article.

## References

[1] Engstrand T., Kihlström L., Neovius E. (2014). Development of a bioactive implant for repair and potential healing of cranial defects. *Journal of Neurosurgery*.

[2] Brie J., Chartier T., Chaput C. (2013). A new custom made bioceramic implant for the repair of large and complex craniofacial bone defects. *Journal of Cranio-Maxillo-Facial Surgery*.

[3] Andani M. T., Shayesteh Moghaddam N., Haberland C., Dean D., Miller M. J., Elahinia M. (2014). Metals for bone implants. Part 1. Powder metallurgy and implant rendering. *Acta Biomaterialia*.

[4] Sundseth J., Sundseth A., Berg-Johnsen J., Sorteberg W., Lindegaard K. F. (2014). Cranioplasty with autologous cryopreserved bone after decompressive craniectomy: complications and risk factors for developing surgical site infection. *Acta Neurochir*.

[5] Klinger D. R., Madden C., Beshay J., White J., Gambrell K., Rickert K. (2014). Autologous and acrylic cranioplasty: a review of 10 years and 258 cases. *World Neurosurgery*.

[6] Honeybul S., Morrison D. A., Ho K. M., Lind C. R., Geelhoed E. (2017). A randomized controlled trial comparing autologous cranioplasty with custom-made titanium cranioplasty. *Journal of Neurosurgery*.

[7] Yang X., Wen L. (2019). Issues in cranioplasty after traumatic skull defect. *Chinese Journal of Trauma*.

[8] Hng D., Bhaskar I., Khan M. (2015). Delayed cranioplasty: outcomes using frozen autologous bone flaps. *Craniomaxillofacial Trauma & Reconstruction*.

[9] el-Deftar M. M. F., el Din Mohamed A., el-Ghannam A. (2017). Regeneration of critical-size canine calvarial defect by silica-calcium phosphate-composite (SCPC) and human adipose derived stem cells. *Key Engineering Materials*.

[10] Lappalainen O. P., Korpi R., Haapea M. (2015). Healing of rabbit calvarial critical-sized defects using autogenous bone grafts and fibrin glue. *Child's Nervous System*.

[11] Peyrin F., Attali D., Chappard C., Benhamou C. L. (2010). Local plate/rod descriptors of 3D trabecular bone micro-CT images from medial axis topologic analysis. *Medical Physics*.

[12] Corliss B., Gooldy T., Vaziri S., Kubilis P., Murad G., Fargen K. (2016). Complications after in vivo and ex vivo autologous bone flap storage for cranioplasty: a comparative analysis of the literature. *World Neurosurgery*.

[13] Wiggins A., Austerberry R., Morrison D., Ho K. M., Honeybul S. (2013). Cranioplasty with custom-made titanium plates–14 years experience. *Neurosurgery*.

[14] Neovius E., Engstrand T. (2010). Craniofacial reconstruction with bone and biomaterials: review over the last 11 years. *Journal of Plastic, Reconstructive & Aesthetic Surgery*.

[15] Camarini E. T., Tomeh J. K., Dias R. R., da Silva E. J. (2011). Reconstruction of frontal bone using specific implant polyether-ether-ketone. *The Journal of Craniofacial Surgery*.

[16] Hanasono M. M., Goel N., DeMonte F. (2009). Calvarial reconstruction with polyetheretherketone implants. *Annals of Plastic Surgery*.

[17] Maugeri R., Giammalva R. G., Graziano F. (2017). Never say never again: a bone graft infection due to a hornet sting, thirty-nine years after cranioplasty. *Surgical Neurology International*.

[18] de Bonis P., Frassanito P., Mangiola A., Nucci C. G., Anile C., Pompucci A. (2012). Cranial repair: how complicated is filling a "hole"?. *Journal of Neurotrauma*.

[19] Frassanito P., Tamburrini G., Massimi L., Peraio S., Caldarelli M., di Rocco C. (2017). Problems of reconstructive cranioplasty after traumatic brain injury in children. *Child's Nervous System*.

[20] Herteleer M., Ectors N., Duflou J., Van Calenbergh F. (2017). Complications of skull reconstruction after decompressive craniectomy. *Acta Chirurgica Belgica*.

[21] Piitulainen J. M., Kauko T., Aitasalo K. M., Vuorinen V., Vallittu P. K., Posti J. P. (2015). Outcomes of cranioplasty with synthetic materials and autologous bone grafts. *World Neurosurgery*.

[22] Lee S. H., Yoo C. J., Lee U., Park C. W., Lee S. G., Kim W. K. (2014). Resorption of autogenous bone graft in cranioplasty: resorption and reintegration failure. *Korean Journal of Neurotrauma*.

[23] Bashaw M. A. (2015). Guideline implementation: autologous tissue management. *AORN Journal*.

[24] Chan D. Y. C., Mok Y. T., Lam P. K. (2017). Cryostored autologous skull bone for cranioplasty? A study on cranial bone flaps’ viability and microbial contamination after deep-frozen storage at −80 °C. *Journal of Clinical Neuroscience*.

[25] Tahir M. Z., Shamim M. S., Sobani Z. A., Zafar S. N., Qadeer M., Bari M. E. (2013). Safety of untreated autologous cranioplasty after extracorporeal storage at -26 degrees Celsius. *British Journal of Neurosurgery*.

[26] Schuss P., Vatter H., Oszvald A. (2013). Bone flap resorption: risk factors for the development of a long-term complication following cranioplasty after decompressive craniectomy. *Journal of Neurotrauma*.

[27] Wu A. C., Raggatt L. J., Alexander K. A., Pettit A. R. (2013). Unraveling macrophage contributions to bone repair. *BoneKEy Reports*.

[28] Li W. M., Han C. L., Liu C., Xing H. Y., Ding D. C. (2018). ANGPTL2 deletion inhibits osteoclast generation by modulating NF- *κ*B/MAPKs/Cyclin pathways. *Biochemical and Biophysical Research Communications*.

[29] Akbar M. A., Nardo D., Chen M. J. (2017). Alpha-1 antitrypsin inhibits RANKL-induced osteoclast formation and functions. *Molecular Medicine*.

[30] Doro D. H., Grigoriadis A. E., Liu K. J. (2017). Calvarial suture-derived stem cells and their contribution to cranial bone repair. *Frontiers in Physiology*.

